# TYRO3 facilitates cell growth and metastasis via activation of the Wnt/β-catenin signaling pathway in human gastric cancer cells

**DOI:** 10.18632/aging.102744

**Published:** 2020-02-04

**Authors:** Dehu Chen, Qinghong Liu, Gan Cao, Wei Zhang

**Affiliations:** 1 Department of General Surgery, Taizhou People’s Hospital, The Fifth Affiliated Hospital of Nantong University, Taizhou 225300, China; 2 Department of Infectious Diseases, Taizhou People’s Hospital, The Fifth Affiliated Hospital of Nantong University, Taizhou 225300, China

**Keywords:** TYRO3, gastric cancer, epithelial-mesenchymal transition, Wnt/β-catenin

## Abstract

It has become increasingly important to identify valuable therapeutic targets to improve the prognosis of cancer patients. Although emerging evidence has suggested TYRO3 as a potential therapeutic target in various types of cancers, less is known about its role in gastric cancer (GC) development. Herein, we investigated the functional and molecular mechanisms by which TYRO3 influenced GC. TYRO3 mRNA and protein were evaluated by quantitative real-time PCR (qRT-PCR), western blotting, and immunohistochemistry. Other methods including stable transfection of TYRO3 into GC cells, wound healing, Transwell assays, CCK-8 assays, colony formation assays, immunocytochemistry *in vitro,* and tumorigenesis *in vivo* were also conducted. Our results indicated that high levels of TYRO3 significantly correlated with clinical metastasis and poor prognoses in patients with GC. In addition, TYRO3 silencing distinctively suppressed GC cell growth, invasion, and metastasis both *in vitro* and *in vivo*. Conversely, TYRO3 overexpression led to the opposite effects. Mechanistic analyses revealed that the Wnt/β-catenin signaling pathway might be involved in TYRO3-facilitated GC cell behavior. Collectively, we demonstrated that elevated TYRO3 expression contributed to GC cell growth and metastasis via the Wnt/β-catenin pathway, suggesting a novel therapeutic target for GC.

## INTRODUCTION

Gastric cancer (GC) is one of the most common causes of cancer-related mortality worldwide [1]. Although considerable progress has been made in surgical treatment and chemotherapy, overall survival (OS) remains poor due to tumor recurrence and metastasis [2]. The detailed mechanisms underlying GC development still remain to be fully elucidated. Further, the increase in the cases of relapse and metastasis has elicited the need for identification of oncogenic and metastases-related biomarkers and the underlying mechanisms driving GC progression.

Recent evidence suggests that epithelial-mesenchymal transition (EMT) is a key event in tumor invasion and metastasis [3]. EMT has also been demonstrated to be involved in other cancer progression events, such as the acquisition of stem cell-like characteristics and resistance to chemotherapy [4, 5]. EMT refers to a transdifferentiation process of epithelial cells into mesenchymal cells, and it is characterized by the loss of the epithelial marker E-cadherin, and the gain of the mesenchymal marker N-cadherin [3]. This switch in cell behavior enhances the ability to accelerate invasion and metastasis, controlled by transcription factors such as Snail, Slug, ZEB1, ZEB2, and Twist, as well as by many different signaling pathways. As previously reported, certain signaling factors involved in EMT include TGF-β, Wnt/β-catenin, PI3K/AKT, Notch, and NF-κB [3, 6, 7]. The interlinked mechanism of cell behavior and EMT has a cumulative effect of augmenting the complexity of the disease, and therefore targeting it is of great importance.

Previous studies have revealed that receptor tyrosine kinases (RTKs) play important roles in cancer progression and have served as ideal therapeutic targets in many types of cancers [8]. To the best of our knowledge, TYRO3 belongs to the TAM (TYRO3, AXL, and MER) receptor family, a unique subfamily of RTKs. Although roles for AXL and MER in cancer progression have been well-described, less is known about the role of TYRO3 [9–10]. Recent studies have indicated that TYRO3 could promote cell proliferation, invasion, and chemoresistance in several human cancers, including bladder cancer [11], colon cancer [12], and breast cancer [13]. However, the biological function and clinical significance of TYRO3 in GC have not yet been elucidated.

In this study, we aimed to investigate the clinical significance and the role of TYRO3 in GC tissues, and the underlying molecular mechanism responsible for the function of TYRO3.

## RESULTS

### Expression of TYRO3 and its significance in patients with GC

**Figure 1:**
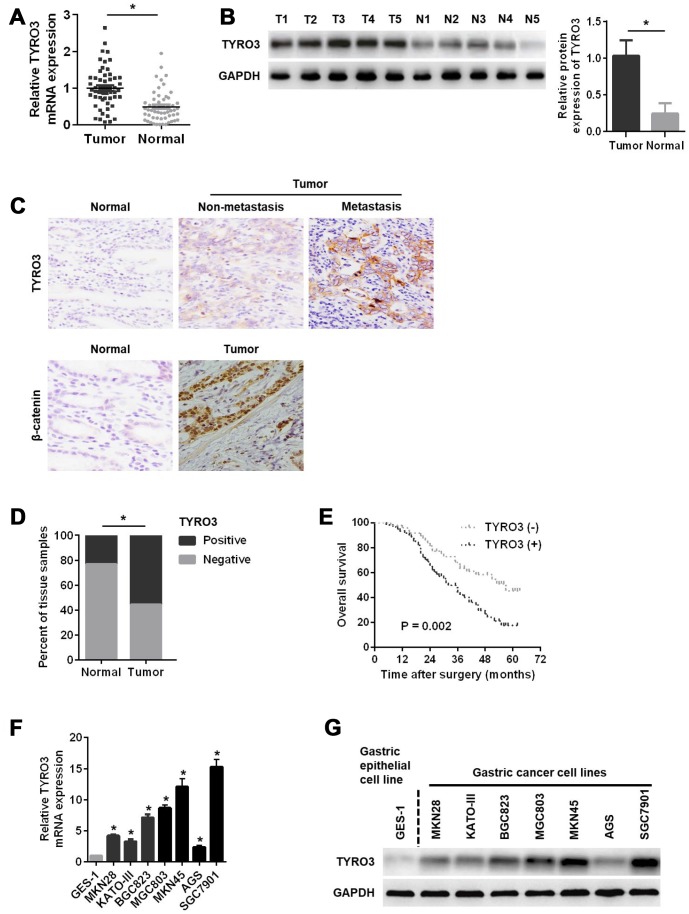
**Relative TYRO3 expression in gastric cancer (GC) tissues and cell lines.** (**A**) The qRT-PCR analysis of TYRO3 mRNA expression in 55 paired specimens of GC tumor tissues versus corresponding normal tissues. (**B**) Western blot analyses of TYRO3 protein expressions in 55 paired specimens of GC tissues versus matched normal tissues. (**C**) Representative images of TYRO3 and β-catenin staining in surgical specimens from 110 paired of GC tissues with or without metastasis and adjacent normal tissues. (**D**) Quantitative evaluation of TYRO3 expression in tumor tissues and matched normal tissues according to staining scores. (**E**) Kaplan-Meier analysis of overall survival in patients with variable expression of TYRO3. (**F**) The qRT-PCR analysis of TYRO3 mRNA expression in GC cell lines and normal gastric epithelial GES-1 cells. (**G**) Western blot analyses of TYRO3 protein expressions in GC cell lines and normal gastric epithelial GES-1 cells. Glyceraldehyde 3-phosphate dehydrogenase was used as a loading control. *P < 0.05.

To elucidate the role of TYRO3 in GC progression, TYRO3 mRNA and protein levels were first examined in 55 pairs of GC tissue samples. As shown in Figure 1A, compared with corresponding normal tissues, the expression levels of TYRO3 mRNA were significantly higher in GC tissues. A similar result was observed for TYRO3 protein analysis using western blotting (Figure 1B). Additionally, immunohistochemical (IHC) analysis of 110 cases further demonstrated that AKIP1, TYRO3, and β-catenin expression levels were significantly upregulated in GC tissues compared with those in matched normal tissues, especially in metastatic GC tissues for TYRO3 (Figure 1C and 1D).

Next, an analysis of the clinicopathological features of 110 GC patients showed that TYRO3 was significantly related to tumor size, T stage, pTNM stage, and lymph node metastasis (*P* < 0.05) (Table 1), suggesting that TYRO3 might play a key role in GC progression and metastasis. Table 2 shows that the expression level of TRYO3 was positively related to that of β-catenin (*P* < 0.001, contingency coefficient = 0.487) in GC tissues. Remarkably, Kaplan-Meier survival analyses indicated that GC patients with higher TYRO3 expression exhibited a shorter OS (Figure 1E).

**Table 1:** Relationship between TYRO3 expression levels and clinicopathological variables in gastric cancer

**Clinicopathological variables**	**n**	**TYRO3**		***P-*value**
		**+**	**-**	
**Age (years)**				
≥60	74	40	34	0.672
<60	36	21	15	
**Sex**				
Male	76	44	32	0.441
Female	34	17	17	
**Tumor size (cm)**				
≥5	69	45	24	0.008
<5	41	16	25	
**Lauren’s classification**				
Diffuse	29	15	14	0.638
Intestinal	81	46	35	
**Lymphatic vessel invasion**				
With	48	29	19	0.357
Without	62	32	30	
**T stage**				
T_1_ + T_2_	51	20	31	0.001
T_3_ + T_4_	59	41	18	
**pTNM stage**				
I + II	49	17	32	<0.001
III + IV	61	44	17	
**Lymph node metastasis**				
With (N_1_+N_2_+N_3_)	67	49	18	<0.001
Without (N_0_)	43	12	31	

**Table 2:** Correlation analysis between TYRO3 expression and β-catenin expression in gastric cancer tissues using the chi-square test

	**TYRO3**				
	**Positive**	**Negative**	**χ^2^**	**P-value**	**C**
**β-catenin**					
Positive	53	16	34.182	<0.001	0.487
Negative	8	33			

Furthermore, the expression levels of TYRO3 mRNA and protein were evaluated in a normal gastric epithelial cell line (GES-1) and GC cell line. Figure 1F and 1G show that both mRNA and protein levels of TYRO3 were higher in GC cell lines than those in GES-1 cells. Taken together, these results implied that TYRO3 overexpression was related to disease progression and poor prognosis in GC.

### Effects of TYRO3 on GC cell growth

**Figure 2:**
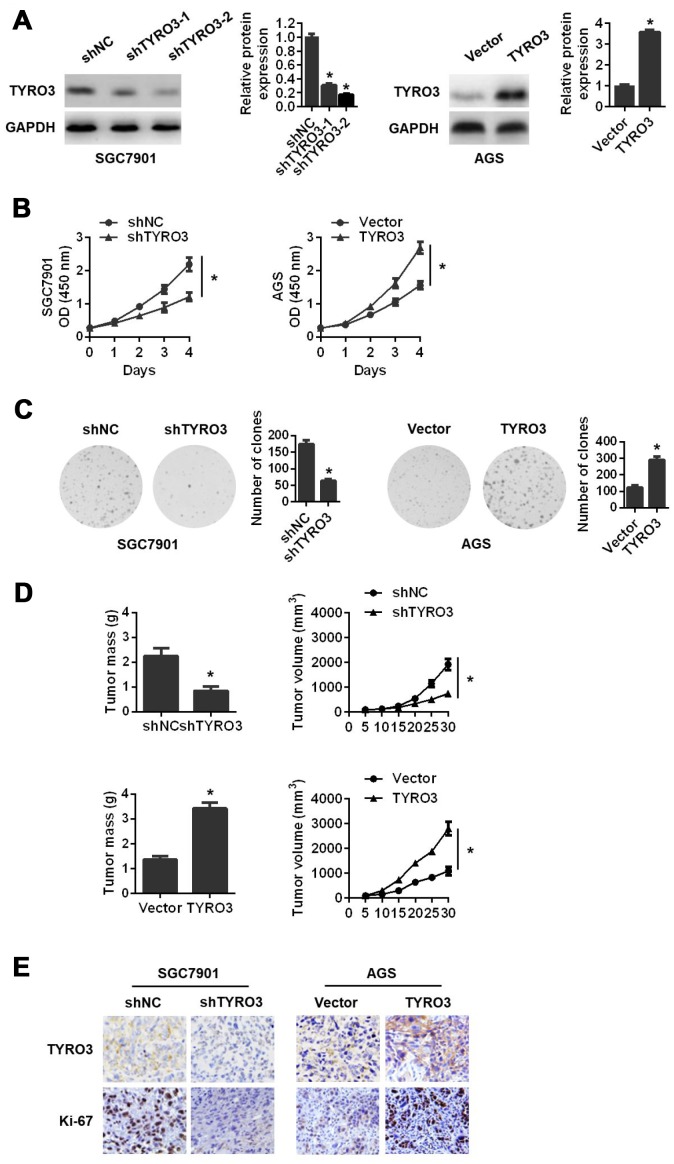
**The effects of TYRO3 knockdown or overexpression on gastric cancer (GC) cell proliferation both in vitro and in vivo.** (**A**) Western blot analyses of TYRO3 expressions in GC cells transfected with the TYRO3-shRNA or TYRO3-constructed plasmid. (**B**) The effect of TYRO3-altered expression on GC cell growth using the CCK-8 assay. (**C**) The effect of TYRO3-altered expression on GC cell growth using the colony formation assay. (**D**) The effect of TYRO3 altered expression on GC cells subcutaneous xenograft growth in nude mice. Tumor mass and tumor volume are shown in the right panels (n = 6/group). (**E**) Immunohistochemical detection of TYRO3 and Ki-67 expression levels in xenograft tumor samples. Glyceraldehyde 3-phosphate dehydrogenase was used as a loading control. *P < 0.05.

To ascertain the effects of TYRO3 on GC cells, we performed gain- and loss-of-function experiments by silencing TYRO3 in SGC7901 cells and overexpressing TYRO3 in AGS cells (Figure 2A). Among two TYRO3 short hairpin RNAs (shRNAs), shTYRO3-2 was selected for subsequent studies because of its more efficient knockdown effect in SGC7901 cells (Figure 2A). The results revealed that TYRO3 knockdown in SGC7901 cells significantly attenuated cell proliferation as determined by the Cell Counting Kit-8 (CCK-8) assay (Figure 2B) and colony formation assay (Figure 2C). Conversely, TYRO3 overexpression in AGS cells led to the opposite results (Figure 2B and 2C).

To further demonstrate the above findings *in vivo*, we established a subcutaneous xenograft tumor model in nude mice. Consistently, we found that TYRO3 knockdown in SGC7901 cells suppressed tumor growth *in vivo*, as evidenced by a reduction of tumor mass and tumor volume (Figure 2D). In contrast, TYRO3 overexpression in AGS cells resulted in the opposite result (Figure 2D). Additionally, IHC analysis of transplanted tumors revealed that TYRO3 knockdown in SGC7901 cells led to a remarkably downregulated expression of Ki-67. As expected, TYRO3 overexpression in AGS cells resulted in a significantly upregulated expression of Ki-67 (Figure 2E). Collectively, these data suggested that TYRO3 had an oncogenic function and facilitated GC cell growth *in vitro* and *in vivo*.

### Effects of TYRO3 on GC cell invasion and metastasis

**Figure 3:**
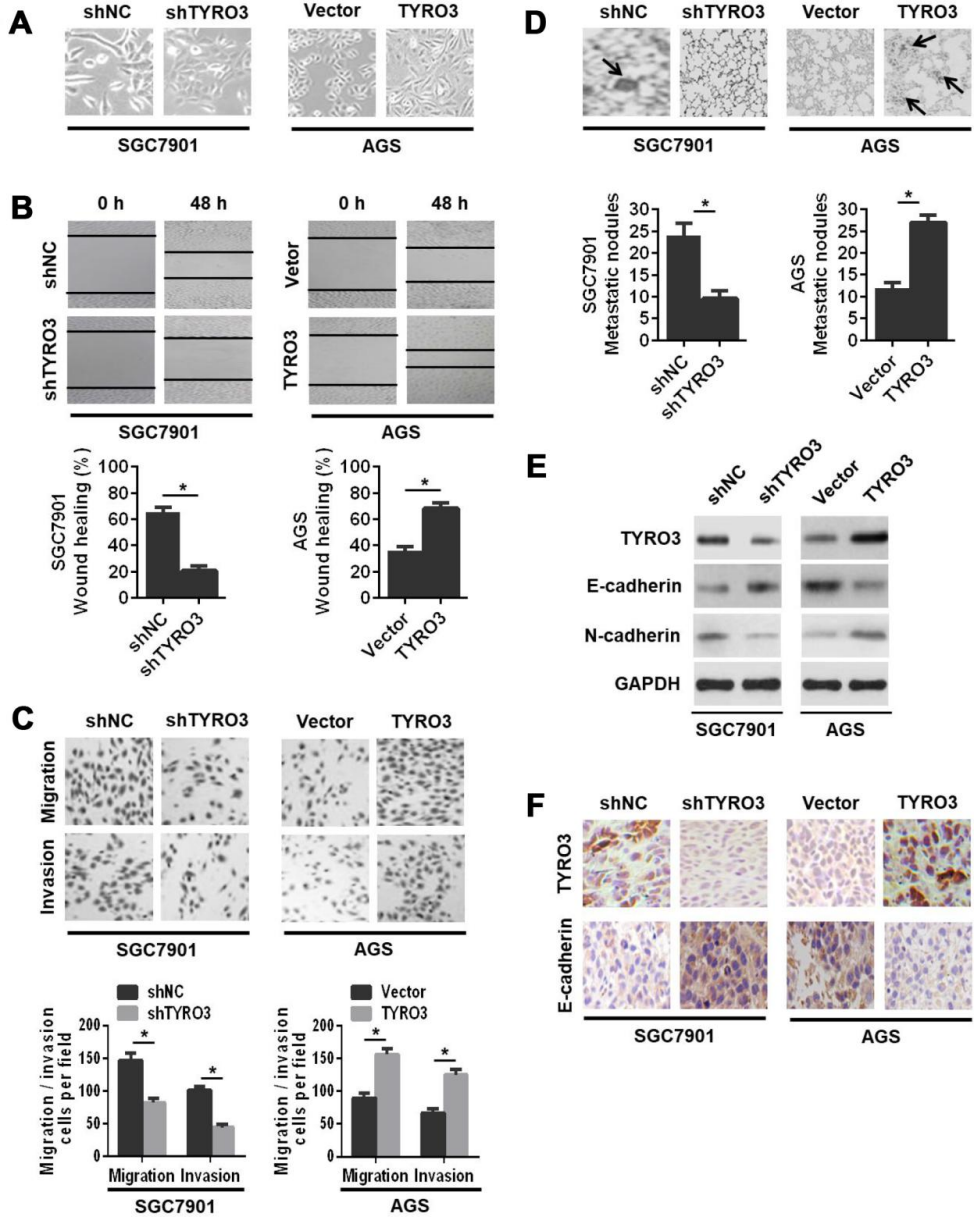
**The effects of TYRO3 downregulation or upregulation on gastric cancer (GC) cell migration and invasion in vitro and metastasis in vivo.** (**A**) Representative images of TYRO3 downregulation or upregulation-induced morphological changes of GC cells. (**B**) The effects of TYRO3 downregulation or upregulation on the migratory capability of GC cells using the wound-healing assay. (**C**) The effects of TYRO3 downregulation or upregulation on the migratory and invasive capabilities of GC cells using the Transwell assay. (**D**) Representative hematoxylin and eosin staining images of lung metastases. Metastatic tumor nodules are indicated by black arrows. (**E**) Western blot analyses of the expression levels of TYRO3, E-cadherin, and N-cadherin in the lung metastatic nodules. (**F**) Immunohistochemical detection of the expression levels of TYRO3 and E-cadherin in the lung metastatic nodules (n = 6/group). Glyceraldehyde 3-phosphate dehydrogenase was used as a loading control. *P < 0.05.

Next, we investigated the role of TYRO3 in GC cell invasion and metastasis. We observed that TYRO3 overexpression in AGS cells induced a morphological change from a cuboidal-like epithelial shape to a spindle shaped fibroblastic phenotype. Conversely, TYRO3 knockdown in SGC7901 cells resulted in a reversal of the morphological changes (Figure 3A). The wound-healing assay showed that TYRO3 overexpression in AGS cells showed a significantly more rapid wound closure; while TYRO3 knockdown in SGC7901 cells led to a slower wound closure (Figure 3B). Consistently, the transwell migration and invasion assays indicated that TYRO3 knockdown significantly suppressed the migration and invasion abilities of SGC7901 cells, whereas TYRO3 overexpression significantly enhanced the migration and invasion abilities of AGS cells (Figure 3C).

To further evaluate the *in vivo* role of TYRO3 in GC cells metastasis, we established a lung metastasis model via the injection of GC cells into the caudal vein in nude mice. The results indicated that TYRO3 knockdown in SGC7901 cells suppressed cell metastasis *in vivo*, as evidenced by a significant decrease in lung metastatic nodules (Figure 3D) and a noticeable reversal of the EMT as shown by western blot analysis (Figure 3E) and IHC analysis (Figure 3F). In contrast, TYRO3 overexpression in AGS cells led to the opposite outcomes (Figure 3D, 3E, and 3F). In summary, these *in vivo* results were consistent with *in vitro* findings, suggesting that TYRO3 facilitated GC cell invasion and metastasis both *in vitro* and *in vivo*.

### The Wnt/β-catenin signaling-mediated EMT is involved in TYRO3-induced GC cell growth and invasion.

**Figure 4:**
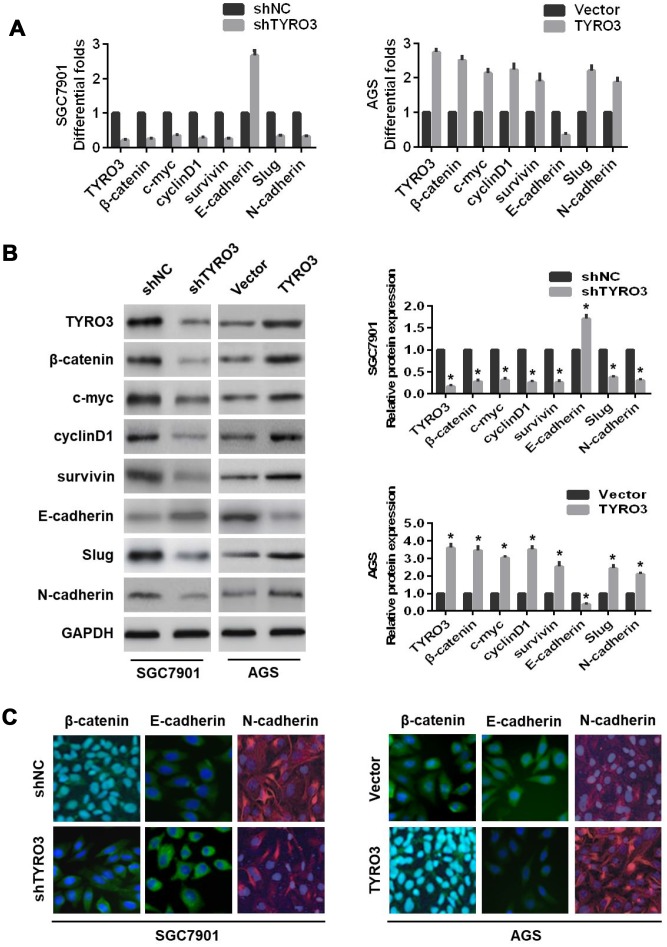
**The effects of TYRO3 knockdown or overexpression on the epithelial-mesenchymal transition and Wnt/β-catenin signaling-related markers in gastric cancer (GC) cells.** Following TYRO3 shRNA or overexpression treatment, (**A**) qRT-PCR, (**B**) western blotting, and (**C**) immunofluorescence detection of the indicated expression levels (TYRO3, β-catenin, c-myc, cyclinD1, survivin, E-cadherin, Slug, and N-cadherin) in modified GC cells. Glyceraldehyde 3-phosphate dehydrogenase was used as a loading control. *P < 0.05.

The β-catenin serves as the key protein of the Wnt signaling pathway, which leads to transcriptional activation of several genes in the EMT pathway [14]. Our study also revealed that TYRO3 expression was positively associated with β-catenin expression in GC tissues. Therefore, we concluded that the Wnt/β-catenin signaling pathway was involved in the TYRO3-mediated EMT in GC. To test this hypothesis, we initially examined the expressions of Wnt/β-catenin signaling-related genes with TYRO3 knockdown or overexpression in GC cells. As shown in Figure 4A and 4B, TYRO3 knockdown in SGC7901 cells resulted in higher levels of E-cadherin and lower levels of β-catenin, the transcriptional targets of Wnt/β-catenin signaling (including c-myc, cyclinD1, and survivin), Slug, and N-cadherin. However, TYRO3 overexpression in AGS cells led to the opposite outcomes (Figure 4A and 4B). Additionally, immunofluorescence staining further confirmed the above findings (Figure 4C).

**Figure 5:**
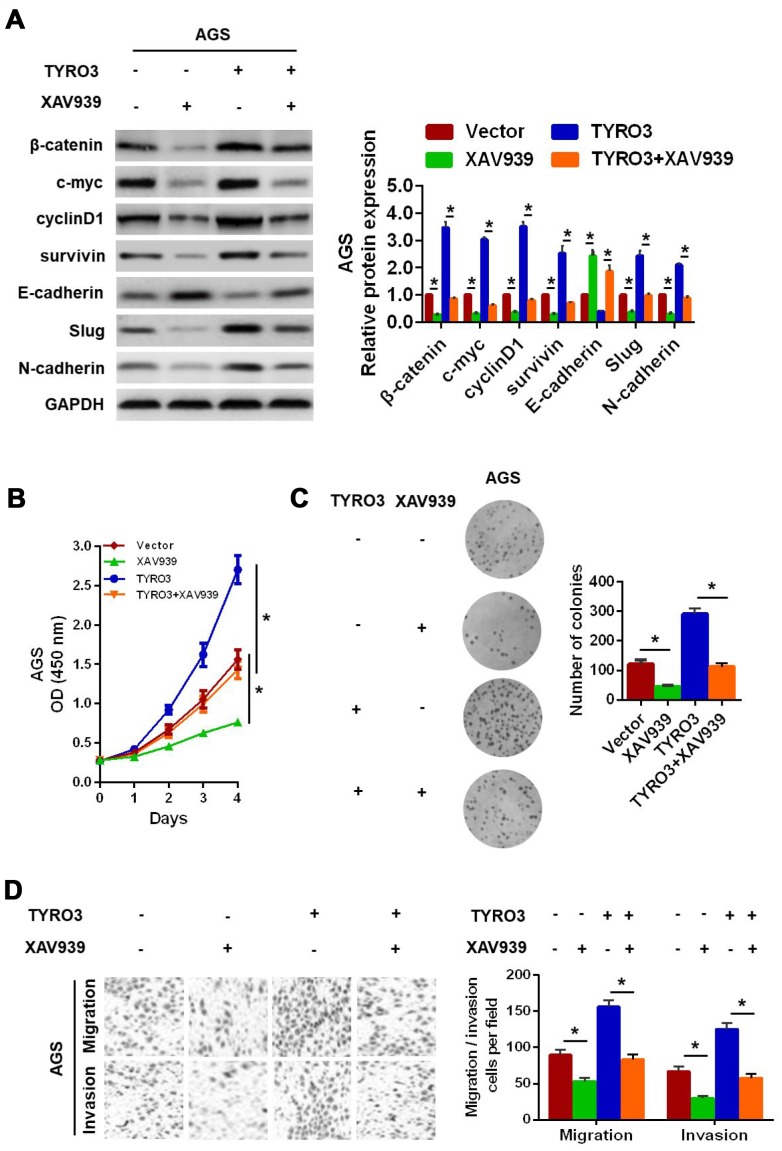
**Wnt/β-catenin signaling involved in TYRO3-facilitated gastric cancer (GC) cell epithelial-mesenchymal transition growth, migration, and invasion.** (**A**) Western blot analysis of the indicated expression levels (β-catenin, c-myc, cyclinD1, survivin, E-cadherin, Slug, and N-cadherin) in modified GC cells treated with the Wnt/β-catenin signaling inhibitor, XAV939 (15 μM). Determination of the proliferative capability of modified GC cells with XAV939 (15 μM) treatment using the CCK-8 assay (**B**) and colony formation assay (**C**). (**D**) Determination of the migratory and invasive capabilities of modified GC cells with XAV939 (15 μM) treatment, using the Transwell assay. Glyceraldehyde 3-phosphate dehydrogenase was used as a loading control. *P < 0.05.

Next, to prove whether the Wnt/β-catenin signaling-mediated EMT was involved in TYRO3-induced GC cell growth and invasion, the specific Wnt/β-catenin signaling inhibitor, XAV939, was used in GC cells. The results showed that XAV939 treatment resulted in the downregulation of the expression levels of the Wnt/β-catenin signaling-related genes (*c-myc, cyclinD1,* and *survivin*) and a remarkable reversal of the TYRO3-induced EMT (Figure 5A) in AGS cells. In addition, XAV939 treatment of AGS cells significantly suppressed TYRO3-facilitated cell growth according to the CCK-8 assay (Figure 5B) and colony formation assay (Figure 5C) results, as well as TYRO3-induced migration and invasion using the Transwell assay (Figure 5D) in AGS cells.

Overall, these results suggested that TYRO3 promoted GC cell growth and invasion via activation of the Wnt/β-catenin signaling-mediated EMT.

## DISCUSSION

Over the past few decades, although significant progress has been made in the treatment of patients with advanced GC, including surgical techniques and chemotherapy, tumor recurrence and metastasis remain the major cause of death in patients with GC [1, 15, 16]. Because of numerous genetic changes responsible for cancer cell growth and metastasis [17, 18, 19], efforts are urgently needed to identify novel key regulators of cancer cell behavior, to expand our understanding of the underlying mechanisms involved in cancer cell evolution for targeted therapies. Notably, emerging evidence has shown that TYRO3 has an important role in cell proliferation, invasion, and chemoresistance in various types of cancers [9]. Notably, a study revealed that TYRO3 knockdown suppressed melanoma cell migration and invasion, while TYRO3 overexpression led to the opposite outcomes via activation of the tyrosyl-phosphorylation of ACTN4, a member of the actin binding protein family involved in cancer cell motility [10, 20]. Evidence has also supported the notion that aberrant expression of TYRO3 contributed to tumorigenesis and metastasis in colon cancer, suggesting TYRO3 as a drug target in cancer therapy [12]. To our knowledge, however, little is known about the clinical significance of TYRO3 in GC tissues, and its molecular functionality and novel mechanism by which TYRO3 influences various physiological processes of GC patients.

In the present study, we discovered that TYRO3 was upregulated in GC tissues compared with that in matched para-carcinoma tissues. Importantly, clinicopathological analysis showed that increased TYRO3 expression was significantly associated with large tumor size, clinical metastasis, and poor prognosis, suggesting that TYRO3 overexpression was strongly suggestive of the proliferative and metastatic state in patients with GC. Next, *in vivo* and *in vitro* findings showed that TYRO3 knockdown resulted in the inhibition of GC cell growth, migration, and invasion; however, TYRO3 overexpression led to the opposite outcomes. Mechanistic analyses demonstrated that TYRO3 promoted cell growth and metastasis through the Wnt/β-catenin signaling-mediated EMT in GC.

Tumor metastasis is a complex and multistage process, and tumor cells must acquire various properties, including altered adhesiveness, increased motility, and invasive capacity, to escape the confines of the primary tumor and establish distant metastases. As well as generating cancer stem cells and contributing to therapy resistance, the EMT process is believed to be implicated in the initial steps of the metastatic cascade by conferring an invasive phenotype [16]. Hence, targeted reversal of EMT may be an effective strategy for treatment of patients with GC.

Herein, an important finding was that the Wnt/β-catenin signaling pathway was a mediator involved in the TYRO3-induced EMT. First, our findings revealed that TYRO3 knockdown reversed the EMT process, whereas TYRO3 overexpression accelerated EMT evolution, suggesting TYRO3 as an important regulator of EMT in GC cells. Second, it is widely accepted that the Wnt/β-catenin signaling pathway has been found to be activated in approximately 30–50% of GC tissues and in several types of GC cell lines [21]. Furthermore, blockade of the Wnt/β-catenin signaling suppresses EMT as well as the proliferation, migration and metastasis of cancer cells [22, 23]. Importantly, it is worth noting that β-catenin is a key protein of the WNT signaling pathway, and β-catenin overexpression could facilitate subsequent transcriptional activation of several genes in the EMT, including c-myc, cyclin D1, and survivin [14]. In the current study, we showed that TYRO3 expression was positively associated with β-catenin expression in GC tissues. Additionally, TYRO3 knockdown in SGC7901 cells decreased the expression levels of the Wnt/β-catenin target genes, whereas TYRO3 overexpression in AGS cells increased their expression levels. Third, to further explore whether TYRO3 facilitates cell growth and metastasis through the Wnt/β-catenin signaling-mediated EMT in GC, the specific Wnt/β-catenin signaling inhibitor, XAV939, was used to conduct studies involving modified GC cells [24]. Our results showed that inhibition of Wnt/β-catenin signaling weakened not only the TYRO3-induced EMT, but also cell growth, migration, and invasion. Taken together, our data suggested that TYRO3 promoted cell growth and metastasis via activation of the Wnt/β-catenin signaling pathway in GC.

Although β-catenin acted as a downstream effector in TYRO3-induced growth, migration, and invasion, it is worth further exploration of the in-depth mechanism(s) involved in the complex interaction of TYRO3 and β-catenin. Research has revealed that β-catenin, a key molecule of the WNT signaling pathway, is a direct or indirect downstream target involved in the aggressive behavior of cancer cells [24]. As reported, RSPO2 suppressed colorectal cancer progression by negatively regulating Wnt/β-catenin signaling through an LGR5-dependent feedback mechanism [25]. Clinically, we found that TYRO3 expression was positively correlated with β-catenin expression. To gain more insight into TYRO3-mediated β-catenin in GC cells, the LGR5-dependent feedback mechanism may be worth further investigation in future studies.

In summary, our current study emphasized that high TYRO3 expression significantly correlated with clinical metastasis and poor prognoses in patients with GC, and TYRO3 promoted cell growth, invasion, and metastasis via activation of the Wnt/β-catenin signaling-mediated EMT, thus possibly providing a potential therapeutic target for GC.

## MATERIALS AND METHODS

### Patients, specimens, and cell lines

GC tissue specimens and homologous non-cancerous tissues were collected from a total of 110 patients who underwent surgical resection at the Fifth Affiliated Hospital of Nantong University. Patients enrolled in this study had not received preoperative chemotherapy or radiotherapy. Written informed consent was obtained from all participants. Our study was approved by the Ethics Committee of the Fifth Affiliated Hospital of Nantong University in accordance with the Declaration of Helsinki.

The normal gastric epithelial cell lines (GES-1 and GC cell lines, including MKN28, KATO-III, BGC823, MGC803, MKN45, AGS, and SGC7901) were obtained from the American Type Culture Collection (Manassas, VA, USA). All cells were routinely propagated in accordance with the manufacturer’s protocols in a 5% CO_2_ humidified incubator at 37°C.

### RNA isolation and qRT-PCR

Total RNA was extracted from tissues or cells using the TRIzol reagent (Thermo Fisher Scientific, Waltham, MA, USA) and reverse-transcribed to cDNA with a reverse transcription reagent kit (TaKaRa, Kusatsu, Japan). The mRNA level was measured using the SYBR Green Assay kit (TaKaRa) and the 7500 RT-PCR detection system (Thermo Fisher Scientific) following the manufacturer's instructions. The mRNA relative expression level was normalized to glyceraldehyde 3-phosphate dehydrogenase (GAPDH), and was quantified by the 2^−ΔΔCt^ method. PCR primer sequences are listed in Supplementary Table 1.

### Western blot analysis

Western blotting was performed according to standard techniques as described previously [18]. Briefly, samples from tissues or cell lysates were separated by SDS-PAGE, transferred onto polyvinylidene difluoride membranes (Millipore, Burlington, MA, USA), blocked with 5% nonfat milk, and then probed with primary antibodies at 4°C overnight, followed by secondary antibody (Sigma-Aldrich, St. Louis, MO, USA) for 2 h at room temperature the next day. The bands were detected with the enhanced chemiluminescence detection reagent (Thermo Fisher Scientific). Primary antibodies against TYRO3, β-catenin, c-myc, cyclinD1, and survivin were obtained from Abcam (Cambridge, UK). Primary antibodies against E-cadherin, Slug, and N-cadherin were obtained from Cell Signaling Technology (Danvers, MA, USA). GAPDH antibody, used as the loading control, was purchased from Bioworld Technology (St. Louis Park, MN, USA).

### IHC and evaluation

IHC analysis was performed as described previously [26]. Briefly, paraffin-embedded tissue sections were deparaffinized, dehydrated, and treated for antigen retrieval. Sections were subsequently blocked by hydrogen peroxide and normal blocking serum, followed by incubation with specific primary antibody overnight. The next day, sections were probed with secondary antibody (Sigma-Aldrich), stained with diaminobenzidine (Boster Biological, Wuhan, China), and scored using a light microscope. Primary antibodies against TYRO3, β-catenin, and Ki-67 were obtained from Abcam. Primary antibodies against E-cadherin and N-cadherin were obtained from Cell Signaling Technology.

The staining scores were graded as described previously [26]. The percentage of staining cells was scored on a scale from 0 to 3 (0 for 0–5%; 1 point for 6–25%; 2 points for 26–50%, and 3 points for 50–100%). The staining intensity was graded as: 0, negative; 1 point, weak; 2 points, moderate; 3 points, strong. Positive IHC expression was defined as the sum of scores ≥ 3 points, while negative IHC expression was considered as the sum of scores < 3 points.

### Transfection

Commercially available TYRO3 shRNA constructs were obtained from Invitrogen (Carlsbad, CA, USA) [13], and the overexpression construct was purchased from OriGene (Rockville, MD, USA) [12]. Scrambled sequence or empty vector was employed as a control. The transfection was processed using Lipofectamine 2000, according to the manufacturer’s instruction. The efficiency of stably transfected cells was evaluated by western blot analysis.

### CCK-8 assay

CCK-8 (Dojindo, Japan) was used to evaluate cell proliferation. Cells (4 × 10^3^) were seeded in a 96-well flat-bottom plate. After culturing for 0, 1, 2, 3, and 4 days, 10 μL CCK-8 solution was added to each well, and further incubated for 80 min. The absorbance at 450 nm was determined for the analysis of cell viability.

### Colony formation assay

Cells were plated in a 6-well plate, stained with crystal violet solution, and counted using a light microscope, after a subsequent culture for 14 days. Clone formation was regarded as those containing ≥ 50 cells.

### Wound-healing assay

Cells were seeded in a 6-well plate and cultured to form a monolayer. A straight wound was then generated by scraping with a 10-μL plastic pipette tip. The wound images were captured at 0 h and 48 h time points to monitor the distance remaining. Wound healing = (0 h width - 48 h width)/0 h width × 100 %.

### Cell migration and invasion assays

Cell migration and invasion assays were conducted as described previously [7]. For the migration assay, cells were seeded in the upper filter of an 8-μm pore size Transwell plate (Corning, Corning, NY, USA) with an uncoated membrane. For the invasion assay, the upper chamber was coated with a matrix gel basement membrane (BD Biosciences, San Jose, CA, USA). In both the migration and invasion assays, medium containing 20% serum as a chemoattractant was added into the lower chamber. After 24 h of incubation, the migratory and invasive cells on the bottom surface of the chamber were fixed with paraformaldehyde, stained with Crystal Violet, and counted using a light microscope.

### Immunofluorescence staining

After being maintained in a 6-well plate for 24 h, cells were fixed with 4% paraformaldehyde and permeabilized with Triton X-100. Subsequently, the cells were blocked with 5% bovine serum albumin and incubated overnight with primary antibodies against TYRO3, E-cadherin, or N-cadherin. The next day, the cells were incubated with Alexa Fluor-conjugated secondary antibody (Bioworld Technology) and then stained with 4′,6-diamidino-2-phenylindole. Finally, the cells were examined and photographed using a fluorescence microscope.

### *In vivo* tumorigenesis and metastasis assays

Nude athymic BALB/c mice (6-weeks-old) were examined in accordance with institutional guidelines, and all procedures were approved by the Ethics Committee of the Fifth Affiliated Hospital of Nantong University. For the tumorigenesis assay, the SGC7901 shNC or shTYRO3 group cells were injected subcutaneously into the flanks of nude mice, followed by measurement of the tumor volume at 5-day intervals using the formula: volume = (short diameter)^2^ × (long diameter)/2 [27]. Thirty days after injection, the mice were sacrificed, and the tumors were harvested for the determination of tumor mass as well as IHC analysis of the expression levels of TYRO3 and Ki-67. For the metastasis assay, the AGS vector or TYRO3 group cells were injected intravenously into the tail vein of nude mice. After 30 days, the lungs of nude mice were removed to count metastatic foci and then subjected to western blot and IHC analyses to determine the expression levels of TYRO3, E-cadherin, and N-cadherin.

### Statistical analysis

Statistical analyses of clinicopathological characteristics were performed using the chi-square method. The Kaplan-Meier and log-rank tests were used for survival analyses. The data were presented as the mean ± SD, and were analyzed by Student's *t-*test or one-way analysis of variance. All statistical comparisons were made using SPSS statistical software for Windows, version 21.0 (SPSS, Chicago, IL, USA). P < 0.05 was considered significant.

## Supplementary Material

Supplementary Table 1
