# Supplementary material for: TYRO3 facilitates cell growth and metastasis via activation of the Wnt/β-catenin signaling pathway in human gastric cancer cells
Source: Aging (Albany NY). 2020 Feb 4;12(3):2261–74. doi: 10.18632/aging.102744 (PMC7041786; doi:10.18632/aging.102744)
Supplement: Supplementary Table 1 [file aging-12-102744-s001..pdf]

## SUPPLEMENTARY TABLE

**Supplementary Table1. Primers designed for qRT-PCR.**

| Genes | Sequence (5'–3') | |
|---|---|---|
| | Forward | Reverse |
| **TYRO3** | GAGGATGGGGGTGAAACC | ACTGTGAAAAATGGCACACCT |
| **β-catenin** | GCTGATTTGATGGAGTTGGA | TCAGCTACTTGTTCTTGAGTGAA |
| **C-myc** | CCTCCACTCGGAAGGACTATC | TGTTCGCCTCTTGACATTCTC |
| **CyclinD1** | GTGCTGCGAAGTGGAAACC | ATCCAGGTGGCGACGATCT |
| **Survivin** | ATGGGTGCCCCGACGTTG | ATGGGTGCCCCGACGTTG |
| **E-cadherin** | CGAGAGCTACACGTTCACGG | GGGTGTCGAGGGAAAAATAGG |
| **Slug** | TTCGGACCCACACATTACCT | GCAGTGAGGGCAAGAAAAAG |
| **N-cadherin** | TCAGGCGTCTGTAGAGGCTT | ATGCACATCCTTCGATAAGACTG |
| **GAPDH** | AGGGGCCATCCACAGTCTTC | AGAAGGCTGGGGCTCATTTG |
